# Histopathological changes induced by paraquat on some tissues of gourami fish (*Trichogaster trichopterus*)

**Published:** 2013-03-30

**Authors:** M. Banaee, M.H. Davoodi, F. Zoheiri

**Affiliations:** *Aquaculture Department, Natural Resource Faculty, Behbahan Khatam Alanbia University of Technology, Behbahan, Iran*

**Keywords:** Histopathology, Gourami fish, Paraquat

## Abstract

Paraquat is a contact and non-selective herbicide which is used for controlling a wide range of terrestrial weeds and aquatic plants. A long-term contact with this xenobiotic can potentially lead to injuries in fishes as live non-target organisms. Therefore, the current study aimed to investigate the effect of sub-lethal toxicity of paraquat on the pathology of gill, liver, and spleen tissues in gourami fish (*Trichogaster trichopterus*). In this study, sub-lethal concentration is determined based on lethal concentration (LC_50_ : 7.16±0.69, 4.46±0.43, 2.19±0.27 and 1.41±0.17 mg/l of paraquat within 24, 48, 72 and 96 hours, respectively). The experiment was done with four varied concentrations of paraquat (0.0, 0.07, 0.15, and 0.3 mg/l equal 0.0%, 5%, 10% and 20% of nominal value of 96 h LC_50_) during 3 weeks. The exposed fish displayed erratic swimming and became lethargic. The changes in gills were characterized by hypertrophy, epithelial, epithelium increase of gill filament, edema and secondary gill lamella. The liver showed hypotrophy of liver cells, cloudy swelling and formation of cytoplasmic vacuoles in the liver tissue of fish treated with 0.15 and 0.3 mg/l concentrations of paraquat. Disorder in the ellipsoid cell and hemosiderin accumulation in melano-macrophage centers was observed in the spleen tissue of fish exposed to 0.15 and 0.3 mg/l of paraquat.

## Introduction

Paraquat (1,1-dimethyl-4,4-bipyridininum ion) which is one of the most common contact and non-selective herbicide for exterminating vegetative pests, is used for controlling terrestrial weeds and aquatic plants in different countries and its presence is reported in many water sources of the world (Filizadeh, 2002; Ye *et al.*, 2002; Gao *et al.*, 2010; Ismail *et al.*, 2011). This herbicide is inactive in soil and is trapped into surface layers of soil via reaction between positive ions of herbicide and negative ions of clay minerals; this issue increases the insolubility of this herbicide in soil (Eizadi-Mood *et al.*, 2011; Ada *et al.*, 2012).

When entering water via microorganisms and under physical conditions, paraquat starts the biological and photochemical decomposition and it disintegrates into methylamine and 4-carboxy-1-methylpyridium ion (Kearney *et al.*, 1985; Eisler, 1990). During the degradation period, via biological accumulation of paraquat in aquatic organisms, especially fishes, macrophytes and algae and its absorption in sediments and floating particles in water, the ground for this toxicant’s influence on non-target organisms is provided (Gabryelak and Klekot, 1985).

The fish contact with paraquat can cause disorders in the activity level of many enzymes involved in cellular biochemical activities, and the change of blood’s biochemical factors, oxidative stress, the change in blood factors and tissue damage. Thus, investigating the toxicity of this herbicide on fishes as one of non-target species seems a necessity for monitoring and assessing the health of aquatic ecosystems.

In fact, photochemical disintegration and auto-oxidation of paraquat can lead to production of products such as hydrogen peroxide, super oxide radical, oxygen and hydroxyl radical which have a great role in creating oxidative stress and the occurrence of cellular toxicity in animal and plant cells (Martin-Rubi *et al.*, 2007). Attacking vital macromolecules, free radicals change the function and nature of their targets and make the ground for many pathological damages (Sureda *et al.*, 2006; Tejada *et al.*, 2007; Banaee *et al.*, 2013a, b).

Due to cell membrane lipid peroxidation of unsaturated fatty acids, short chain fatty acids with R-COOH, R-OOH, R-CHO, and R-OH bases are created which seriously affect the cellular membrane functions such as the activity of hormone receptors and neural mediators, ion transport channels and the activity of membrane enzymes and the transportation of specific molecules. On the other hand, the formation of malondialdehyde (MAD) during peroxidation process of fatty acids having double bonds can create covalent bonds and polymerize cellular membrane components. The propagation of malondialdehyde into cells can make the ground for its reaction with nitrogen alkalis of DNA strands (Sureda *et al.*, 2006; Tejada *et al.*, 2007). Thus, the probability of genetic mutation and even tumor creation increases in animals exposed to paraquat herbicide (Kelley and Van Beneden, 2000).

Due to having unsaturated and sulphuric molecules, amino acids such as phenylalanine, mitonin, cysteine, histidine, and tryptophan are increasingly sensitive and vulnerable to free radicals and specifically reactive oxygen species (Sureda *et al.*, 2006; Tejada *et al.*, 2007) and it helps breaking the sequence of amino acids, aggregation of amino acid chains and even changing the biochemical structure of amino acids and it leads to proteolytic changes in protein compounds (Asada and Barba, 2004). Therefore, damage to protein structures of the tissue and also lipid structure of cell membrane can show itself as tissue damage.

Histopathological indices can be one of the tools in studying the refinement and evaluating the effects of this herbicide on the fish. So, this biological index can be useful in providing an appropriate laboratory pattern (Rabitto *et al.*, 2005; Banaee *et al.*, 2013a; Banaee *et al.*, 2013b). The aims of the present study were to determine the median lethal concentration (96 h LC_50_) of paraquat and to study the histopathological changes in the tissues of gill, liver, and spleen of gourami fish (*Trichogaster trichopterus*) in sub-lethal paraquat exposure. Choosing gourami as a laboratory model is because of the unique features of different gourami species, which have been used in toxicological studies (Hedayati *et al.*, 2012). This fish can easily adjust to laboratory conditions and is highly tolerant to environmental changes.

## Materials and Methods

### Fish

The adult ornamental gourami fish (*Trichogaster trichopterus*) with body weight of 7.15±2.12 g and total length 8.45±0.69 cm were purchased from local fish dealer and used as laboratory models. They were placed in the 85 L aquarium and acclimatized in the laboratory in dechlorinated tap water at 24±2°C with natural photoperiod (16L:8D), pH: 7.2±0.2 and total hardness 280±20 mg/L for 15 days prior to use. The fishes were fed with commercial food twice a day.

### Acute toxicity test

The acute toxicity test was performed according to semi-static methods described in the OECD procedure. The fishes were not fed 24 h before the experiments and during the acute toxicity test. The experiments consisted of a control group and five experimental groups. Acute test was performed to determine the appropriate toxicity range. Assuming acute toxicity, 10 fishes per group were exposed to different paraquat concentrations (0.0, 0.5, 1.5, 3, 6 and 9 mg/L paraquat, purity 30%) in 85L aquarium. During the 96 h acute toxicity experiment, water in each aquarium was aerated and had the same conditions as the acclimation period. Test solutions were renewed every 24 h to maintain the chemical and the water quality. Every 24 h the dead fishes were removed and the numbers of survivals were recorded. The experimental was repeated in triplicate. LC_50_ values were calculated by the Probit Analysis test (Aydın and Köprücü, 2005).

### Sub-lethal toxicity test

For sub-lethal toxicity tests, the concentrations of paraquat in water were maintained modestly below the 96 h LC_50_ value. Based on this value, three sub-lethal concentrations 5%, 10% and 20% of 96 h LC_50_ was chosen for the gourami fish (*T.trichopterus*). The fishes were divided into three treatments and control group by triplicate (10 fishes per each aquarium). Test solutions in each aquarium were renewed every 24 h. On the other hand, the twenty percent of water was changed daily to reduce the build-up of metabolic wastes and to keep concentrations of paraquat near the nominal level. During the experiment, fishes were fed twice a day with commercial food, and mortality was recorded. At the end of 21 days, fishes were sacrificed and organs were obtained and prepared for histopathological and biochemical analysis.

### Tissue histopathological analysis

In the end of experiment periods, on 21 days, fifteen fish per treatment were captured and sacrificed. Then, they were dissected and tissues were collected and washed by buffered normal saline. Tissues were fixed into bouin’s solution (prepared with saturated picric acid, formaldehyde and acetic acid), for 48 h and then dehydrated through graded alcohol series (70 to 100%), cleared in xylene and embedded in paraffin. 5 to 6 µm thick paraffin sections were cut and stained with haematoxylin-eosin and investigated and analyzed under a light microscope (XSZ-801BN model, China) equipped with 12.1 mega pixels’ camera (Casio, EX-Z450, Japan); (Banaee *et al.*, 2013a, b).

## Results

The calculated value of LC_50_ at 24, 48, 72, and 96 hours after starting the test is presented in Table 1. Based on the results LC_50_ values significantly decreased in accordance with the exposure time from 7.16±0.69 mg/L at 24 h to 1.41±0.17 mg/L at 96 h.

**Table 1:** Median Lethal Concentrations of paraquat to gourami

Duration of exposure (h)	LC_50_ (mg/L) value
24	7.16±0.69 (6.01-8.94)
48	4.46±0.43 (3.67-5.40)
72	2.19±0.27 (1.67-2.76)
96	1.41±0.17 (1.07-1.78)

Sub-lethal concentrations of paraquat (0.07, 0.15, and 0.3 mg/l) were equivalent to approximately 5%, 10% and 20% of 96 h LC_50_ value for 21 day toxicity testing. Therefore, no mortality was recorded during the experimental period for all treatment groups studied. In some cases, fish exposed to paraquat exhibited vertical and downward swimming patterns, swimming near the water surface, lethargic and erratic swimming, loss of schooling behaviour, hyperactivity, loss of buoyancy, increased cough rate, increased gill mucus secretions, flaring of the gill arches. The change of body’s color pattern of fish, bleeding around the eyeball and the base of pectoral fins were observed in fish exposed to 0.15 and 0.3 mg/l of paraquat.

**Fig. 1:**
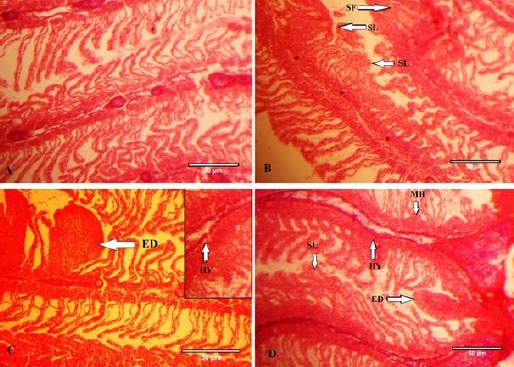
Gills of gourami fish from the control group (Figure A); Gills of gourami fish exposed to 0.07, 0.15 and 30 mg/L of paraquat showing crusting and necrosis of secondary lamellae’s epithelium (Figure B, C & D), mucosa cell hyperplasia (MH), clubbing tips of gill filaments (SF); Changes in cartilage tissue of the gill filament (Figure C & D), fusion of secondary lamellae (SL), edema (ED) and epithelial hyperplasia, the loss of the secondary filaments; (Magnification of the sections 400X).

**Fig. 2:**
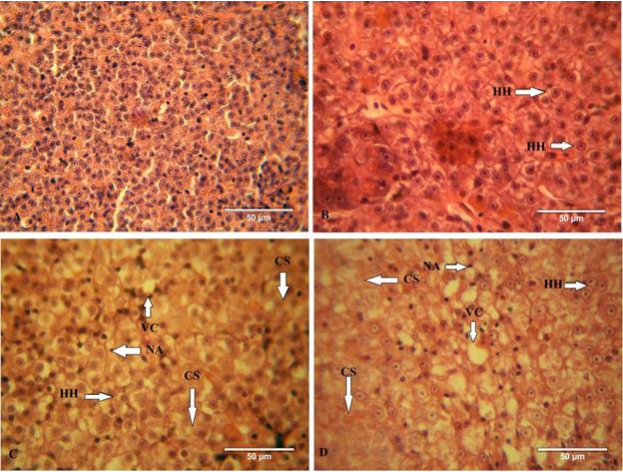
(Figure A) Liver of control fish showing hexagonal hepatic cells (HC) surrounded with the sinusoidal portal blood; (Figure B & C) Liver of 0.07 mg/L and 0.15 mg/L of paraquat-treated fish showing increased hypertrophy of hepatocytes (HH), vacuolization of cell cytoplasm (VC), hepatocyte cloudy swelling (CS); and at the same time hepatocytes lost their normal polygonal structure. Liver of 0.30 mg/L of paraquat-treated fish showing increased cellular degradation with cytoplasm vacuolization (VC) and nucleus atrophy (NA); (Figure D). The hepatic cells form clusters; hypertrophy (HH) and the disorientation in the hepatocytes and bile duct obstruction, hepatocyte cloudy swelling (CS), are found to be more prominent; (Magnification of the sections 400X).

**Fig. 3:**
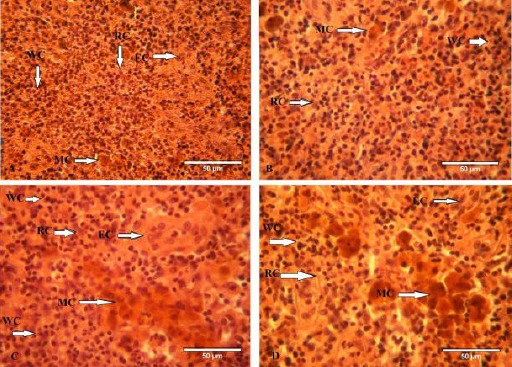
Spleen of gourami fish from the control group (A): With pulp (WP), Red Pulp (RP); Spleen of gourami fish exposed to 0.07 mg/L and 15 mg/L paraquat (Figure B & C): Melanomacrophage centers (MC), Ellipsoid cells (EC), Expansion of splenic red pulp and melano-macrophage centers, disorientation in ellipsoid cells; Spleen of gourami exposed to 0.30 mg/L paraquat (Figure D): Disorder in ellipsoid cells, increase the number and size of melano-macrophage centers, cloudy swelling in spleen tissue; (Magnification of the sections 400X).

Morphological lesions observed in gill, liver and spleen of gourami, *T.trichopterus*, revealed important alterations throughout the experiment. Tissue damages and injuries after 21-day paraquat exposure are illustrated in Figure 1-3. Individuals in the control group did not display any histological changes in any of the examined tissues.

Fish exposed to paraquat showed hyperplasia of gill lamellae and increased gill lamellae thickness resulting in fusion and necrosis. Hyperplasia of the epithelial lining of the secondary lamellae, necrosis and shortening of the secondary lamella, abnormal raising or swelling of the epithelium, as well as fusion of the secondary lamellae, edematous changes and excessive mucus secretion, were observed in fish exposed to paraquat (Fig. 1). These changes intensify with the increase of paraquat concentration.

Liver size relatively increased in all fish exposed to paraquat. Liver cells of fish exposed to 0.07 mg/l paraquat had mild necrosis with occasional diffuse vacuolar degeneration of other hepatocytes. Fish exposed to 0.15 and 0.30 mg/l concentrations of paraquat had seriously necrosis of the liver tissue. Degeneration of hepatocytes with cytoplasm vacuolation and hypertrophy were also noticed. Bleeding and the increase of the liver tissue is one of the microscopic changes observed in these fishes (Fig. 2).

Expansion of splenic white pulps was characterized by prominent vascular congestion and the increase of leukocyte centers at 0.15 and 0.30 mg/l concentrations of paraquat when compared to the control group. Disorder in ellipsoid cells and deposition of hemosiderin in melano-macrophage centers were also seen in spleen’s tissue of fish treated by paraquat (Fig. 3).

## Discussion

Based on the findings of this study, 24, 48, 72, and 96 h LC_50_ values for paraquat in the gourami fish were 7.16±0.69, 4.46±0.43, 2.19±0.27 and 1.41±0.17 mg/l respectively. However, based on a report provided by the United States Fish and Wildlife Service (FWS) in Maryland, value of 96 h paraquat in killfish (*Fundulus similis*), gambusia (*gambusia affinis*), Zebra fish (*Brachydanio rerio*), molly (*Poecilia mexicana*), medaka (*Oryzias latipes*), bluegill fish (*Lepomis macrochirus*), guppy (*Poecilia reticulata*), rainbow trout (*Oncorhynchus mykiss*), brown trout (*Salmo trutta*), channel catfish (*Ictalurus punctatus*) are 1, 3, 60, 7.5, 12, 13, 15, 25, and more than 100 mg/l respectively (Eisler, 1990). The sensitivity of varied species and probably varied environmental conditions can be the most important factor for such a significant difference in the LC_50_ values for paraquat for varied species of fish.

Gill is one of the most important organs directly in contact with pollutants and any kind of damage to the gill tissue of fish leads to disorder in the gas exchange process and also the decrease of ion regulation efficiency via this organ (Ajani *et al.*, 2007). Histopathology of gill is the appropriate bio-indicator to pollution monitoring (Nero *et al.*, 2006a; Nero *et al.*, 2006b; Banaee, 2010; Banaee, 2012).

Our results indicated that the major alterations in the gills of gourami exposed to 0.15 and 0.30 mg/l paraquat were cellular hypertrophy, mucosal cell hyperplasia, fusion of the secondary lamella, sticking of gills filaments, vacuolization, and gills epithelial necrosis, increase of mucosal secretion and edematous.

Gill histopathological damage was also observed after exposure of mosquitofish (*Gambusia affinis*) to deltamethrin (Cengiz and Unlu, 2006), yellow perch and (*Perca flavescens*), goldfish (*Carassius auratus*) and bluegill fish (*Lepomis macrochirus*) and carp (*Cyprinus carpio*) to oil sands (Nero *et al.*, 2006b), yellow perch (*Perca flavescens*) to naphthenic acid (Nero *et al.*, 2006a), carp (*Cyprinus carpio*) to deltamethrin (Cengiz, 2006), tilapia to glyphosphate (Ayoola, 2008), and rainbow trout (*Oncorhynchus mykiss*) to maneb, carbaryl (Boran *et al.*, 2010) and diazinon (Banaee, 2010; Banaee *et al.*, 2011) and deltamethrin insecticide (Cengiz, 2006) respectively. Besides the aforementioned tissue damages, some researchers report chronic respiratory disorders due to the toxic effect of paraquat on the gills of fish (Omitoyin *et al.*, 2006; Ladipo and Doherty, 2011).

There are records of similar changes in the gill tissue of (*Prochilodus lineatus*) (Simonato *et al.*, 2008), carp (Sepici-Dincel *et al.*, 2009), and rainbow trout (Isik and Celik, 2008) which were exposed to varied xenobiotics. Damages incurred to the gills of fish treated with paraquat might be due to oxidative damages to the cells of this tissue. Because, reactive oxygen species (ROS) produced due to degradation and metabolism of pollutants in the environment and body of fish leads to disorder in physical activity of these cells such as ion regulation and gas exchange (Koksoy, 2002).

Histologically, similar to other fish, the liver cells of gourami has a hexagonal appearance with a round nuclei and a monotonous cytoplasm. Bleeding and size increase of liver tissue are among the most important microscopic changes observed in these fishes.

Histopathological investigation indicated important alternations in the liver tissue including necrosis, and changes in nuclear shape and heterochromatin distribution, cellular disarrangement, the formation of vacuoles and fat accumulation in tissue structure of liver and the atrophy of hepatocyte cells in the fish treated with 0.15 and 0.3 mg/l of paraquat is conspicuous.

Similar histopathological changes are reported in the liver tissue of *C.punctatus*, *Gambusia affinis*, *O.mykiss* and *Cyprinus carpio* after exposure to arsenic, deltamethrin, diazinon and heavy metal, respectively (Roy and Bhattacharya, 2006; Cengiz and Unlu, 2006; Banaee *et al.*, 2012b; Vinodhini and Narayanan, 2009). This result is similar to the observations by Cattaneo *et al*. (2008) and Ladipo and Doherty, (2011) in the liver of African catfish (*Clarias gariepinus*) and silver catfish, (*Rhamdia quelen*) to paraquat and dichlorophenoxiacetic acid (2,4-D) herbicide exposure, respectively.

Necrosis and structure destruction of liver cells in these fishes clearly shows the effect of paraquat in destroying the cellular membrane and so necrosis of liver cells. In other words, liver is a place for multiple oxidation reactions and producing the most free radicals in body. Therefore, there is this probability that due to lipid peroxidation, the cellular membrane destroy and the activity of ion regulation channels in the cellular membrane level, especially calcium and other ions disturb and it inhibits intracellular oxidative phosphorylation (Zaragoza *et al.*, 2000).

Due to disturbance in cellular and osmotic regulation power of cellular and biological membranes, the volume of the nuclei and nucleoli increase and it leads to necrosis of liver cells (Ahmad *et al.*, 2002). Figueiredo-Fernandes *et al*. (2006) and Ada *et al*. (2012) showed the influence of paraquat on tilapia (*Oreochromis niloticus*) and its effects on liver parenchyma tissue such as vacuolation, cell necrosis, the increase of macrophage centers, and the accumulation of eosinophil cells.

Hematopoietic centers are one of the most important animal tissues which face unfavorable consequences because of toxicants. Spleen is one of the most important hematopoietic centers.

A significant deposition of hemosiderin granules in a melano-macrophage center in spleen of gourami after exposure to 0.15 and 0.3 mg/l paraquat concentrations might indicate the accumulation of iron containing proteins such as hemosiderin and ferritin in the spleen of this fish. Disorganization of ellipsoid cells and the increase of leukocyte centers are among the shape changes in the spleen tissue of fish treated with paraquat.

Therefore, the size and quantity increase of melano-macrophage centers, especially in spleen, disorientation of ellipsoid cells and resizing white pulp can be one of the major biological indices used in diagnosing stressful conditions (Schwindt *et al.*, 2006; Banaee *et al.*, 2011).

These results are similar to the 3,4-dichloroaniline and captanin effects that have been described in common goby (*Pomatoschistus microps*) and rainbow trout, respectively (Monteiro *et al.*, 2006; Boran *et al.*, 2012). Similar histopathological changes are reported in the spleen of *Notropis hudsonius* (Pulsford *et al.*, 1994), *O.mykiss* (Banaee *et al.*, 2012a) and *Limanda limanda* (Thilakaratne *et al.*, 2007) exposed to environmental pollution.

Explaining the mechanism of histological damages incurred to the tissue of fish might be associated with hydrogen peroxide and superoxide radicals’ function and after disintegrating into destructing hydrogen radicals, necessary conditions for oxidation of organic material is provided (Asada and Barba, 2004). Extensive pathological changes in the tissue of liver, spleen, and gill of fish treated with paraquat can disturb homeostasis and lead to physiological disorders in these animals. Sometimes, the damage incurred to varied tissues is so severe that failure or varied disorders in their function can lead to the death of the fish.
